# Assessing subcortical, brainstem and cerebellar metabolic patterns using [18F]FDG PET-CT imaging in dementia with Lewy bodies

**DOI:** 10.1007/s00259-026-07925-z

**Published:** 2026-06-08

**Authors:** Daniël S. L. Loewenstein, Max van Grinsven, Cécile de Pont, Anne I. J. Arens, Paul L. J. Dautzenberg, Astrid M. van Strien, Osama Sabri, Swen Hesse, Michael Rullmann, Dylan Henssen

**Affiliations:** 1 Department of Medical Imaging, Radboud University Medical Center, Geert Grooteplein Zuid 10, 6525 EZ Nijmegen, The Netherlands; 2 Department of Medical Imaging, Jeroen Bosch Hospital, ‘s Hertogenbosch, the Netherlands; 3 Department of Geriatrics, Jeroen Bosch Hospital, ‘s Hertogenbosch, the Netherlands; 4 Department of Nuclear Medicine, Leipzig University Hospital, Leipzig, Germany

**Keywords:** Dementia with Lewy bodies, FDG PET, Neurodegenerative disorders, Neuroimaging, Dentatorubrothalamic tract, Superior cerebellar peduncle, Tectum mesencephali, Superior colliculus

## Abstract

**Purpose:**

Numerous clinical features of Dementia with Lewy Bodies (DLB) are attributed to dysfunction in subcortical anatomy. Despite this, [^18^F]FDG PET imaging as a diagnostic tool for DLB largely relies on the metabolic signature of the occipital lobe, precuneus, and posterior cingulate cortex. This study aimed to assess subcortical brain metabolism in patients with DLB using [^18^F]FDG PET imaging.

**Methods:**

Patients diagnosed with probable DLB were included from both a prospectively maintained regional database (*n* = 33), and the ADNI database (*n* = 43). Using statistical parametric mapping (SPM) analysis, metabolic activity was compared with a cohort of subjects exhibiting normal brain metabolism (*n* = 19). A sub-analysis was conducted with disease progression included as a covariate.

**Results:**

Hypermetabolism was observed in various subcortical regions, notably in the dentate nucleus, anterolateral thalamus, and regions of the superior cerebellar peduncle. Increased metabolism was also detected in the mesencephalic tectum, likely representing heightened activity in the superior colliculus. All findings were reproduced in the ADNI cohort and were found to be dependent on the DLB disease stage. Additionally, the well-established cortical hypometabolic signature of DLB pathology was evident, validating our methods and findings.

**Conclusion:**

Increased metabolic activity is evident in a variety of brainstem, cerebellar, and subcortical regions in patients with DLB. The dentatorubrothalamic tract, in particular, emerges as a structure of interest that connects these structures and potentially helps in understanding DLB pathophysiology. Correction for disease stage eliminated this pattern, suggesting a driver associated with disease progression.

**Supplementary Information:**

The online version contains supplementary material available at https://doi.org/10.1007/s00259-026-07925-z.

## Introduction

The prevalence of dementia is rising rapidly, with an expected increase of almost 100% every 20 years, making this one of the most urgent challenges in medicine [1]. Neurodegenerative dementia has many causes, with Dementia with Lewy Bodies (DLB) being the second most common type worldwide, accounting for 15–20% of all dementia cases [2, 3]. Disease onset and progression are mediated by the aggregation of the presynaptic neuronal protein α-synuclein. Despite recent research on α-synuclein blood analysis, more work is needed to incorporate it into routine clinical practice [4]. As such, α-synuclein pathology can only be definitively identified through immunohistochemical staining of brain tissue, necessitating post-mortem examination for confirmation [5, 6]. The current diagnostic approach relies predominantly on a characteristic clinical triad of symptoms: 1) fluctuating cognitive impairment, 2) recurrent visual hallucinations, and 3) parkinsonism. However, the degree to which these symptoms are displayed and their clinical onset differ considerably between individuals, hindering diagnosis [7–9]. Differentiation between etiologies of dementia can be aided by PET imaging using [^18^F]Fluorodeoxyglucose ([^18^F]FDG) to assess brain metabolism. Although different causes of dementia show different patterns of hypo- and hypermetabolism, they also affect overlapping brain regions. This makes distinguishing between diagnoses substantially more difficult and impacts diagnostic sensitivity and specificity [7].

Besides DLB, other dementia causes can also affect posterior brain regions such as the dorsal parietal lobes and the occipital lobes, with posterior cortical atrophy (PCA)– an atypical form of Alzheimer’s Dementia – being the most well-known cause. Although posterior cortical atrophy and DLB are differentiable by the use of a few cortical metabolic signs (i.e. metabolic pattern of spread, cingulate island sign, occipital pole sign, and occipital tunnel sign), overlap occurs and misdiagnoses are prevalent [10, 11]. However, the metabolic patterns of subcortical structures in DLB remain largely elusive, even though the pathology underlying important features is believed to be attributed to dysfunction of subcortical regions [12–17]. Identifying subcortical metabolic patterns grounded in disease-specific pathophysiology may therefore provide valuable biomarkers for differentiating DLB from PCA. Therefore, this study characterizes changes in glucose metabolism of subcortical structures, the brainstem, and the cerebellum in patients with DLB by using [^18^F]FDG -PET imaging.

## Materials and methods

### Ethical approval

The regional ethical review board (METC Brabant) waived the requirement for ethical approval given the retrospective nature of this study. Patients who had not provided consent for the utilization of their imaging data in scientific research were excluded from the study.

### Patient selection

Retrospective inclusion took place from a prospectively kept clinical database from Jeroen Bosch Hospital, ‘s-Hertogenbosch, The Netherlands. Patients who underwent [^18^F]FDG PET brain imaging between 2016 and 2022 were selected. For validation, a second cohort was created using patient data from the Alzheimer’s Disease Neuroimaging Initiative (ADNI) database [18]. Here, patients who underwent [^18^F]FDG PET brain imaging between 2006 and 2020 were selected.

In our prospectively kept, local database, two groups of patients were eligible for inclusion in this study: a) patients with no abnormal brain metabolism as assessed by a board-certified nuclear medicine physician; b) patients with metabolic patterns and clinical characteristics of DLB.

Exclusion criteria consisted of: 1) patients younger than 18 years; 2) patients who did not provide consent to share anonymized retrospective data in the context of research purposes; 3) patients who showed metabolic patterns on [^18^F]FDG PET suggestive of other neurodegenerative disorders (e.g., Alzheimer’s disease, Frontotemporal dementia, Corticobasal Degeneration); 4) no clear final clinical diagnosis; and 5) insufficient PET data (e.g., significant motion artefacts). Definitive diagnoses for all patients were based on a combination of clinical parameters, disease progression over time, and [18F]FDG-PET findings. Moreover, all patients underwent dopamine transporter scintigraphy, yielding abnormal results in every case. In many cases, the final diagnosis could be established in the period after the FDG-PET scan used in this study, allowing additional clinical features to emerge and become more pronounced over time. This enabled a diagnosis of DLB to be made with greater certainty. Cases in which diagnostic doubt persisted were not included.

Brain [^18^F]FDG PET imaging was performed 30 min after injection of an average of 165 MBq [^18^F]FDG. Blood glucose levels were checked prior to [^18^F]FDG administration. When hyperglycemia was present (defined as > 160 mg/dl; > 8.9 mmol/L) the imaging session was rescheduled. In accordance with EANM procedure guidelines, a resolution of a 6.5 mm full-width at half-maximum (FWHM) was used for all [^18^F]FDG PET imaging [19]. The PET camera was set in three-dimensional mode for improved sensitivity and the PET camera detectors were positioned over the patient’s head, perpendicular to the orbital-meatal line. Patients were instructed to keep their heads still during image acquisition, since movement of the head would interfere with image quality and compromise the validity of the attenuation correction algorithm. All brain PET imaging was performed on a Siemens Biograph mCT scanner (Siemens Medical Solutions, Hoffman Estates, IL, USA). Low-dose CT was performed with the patient in the same bed position as for PET and the acquired data were used for attenuation correction as well as anatomic correlation.

Data used in the preparation of this article were obtained from the Alzheimer’s Disease Neuroimaging Initiative (ADNI) database (adni.loni.usc.edu). The ADNI was launched in 2003 as a public–private partnership, led by Principal Investigator Michael W. Weiner, MD. The primary goal of ADNI has been to test whether serial magnetic resonance imaging (MRI), positron emission tomography (PET), other biological markers, and clinical and neuropsychological assessment can be combined to measure the progression of mild cognitive impairment and early Alzheimer’s disease. ADNI acquisition procedures have been described in detail elsewhere (https://adni.loni.usc.edu/help-faqs/adni-documentation/). In summary, [^18^F]FDG PET images were acquired using a range of scanners, consisting of ECAT HR +, General Electric Discovery LS, General Electric Discovery ST, General Electric Discovery STE, Siemens/ECAT HRRT, Siemens Biograph Hi-Rez, Philiphs Gemini TF, Siemens mCT and ECAT Biograph.

In the ADNI validation cohort, definitive diagnoses for all patients were established by cerebral autopsy, the gold standard, and all cases showed post-mortem confirmed α-synuclein pathology. Disease stages per patient were classified according to the modified McKeith criteria (3): (1) Brainstem predominant; (2) Limbic (transitional); (3) Neocortical (diffuse); (4) Amygdala predominant; (5) Olfactory bulb.

Demographic details about all patients are presented in Table 1.

**Table 1:** Demographic characteristics of participants

Participant number	Sex	Year of Birth	Age at Scan	Age difference (Autopsy – Scan)	Disease Stage at Autopsy
*JBZ*					
JBZ_001	F	1946	74	-	-
JBZ_002	M	1947	72	-	-
JBZ_003	M	1951	69	-	-
JBZ_004	M	1947	73	-	-
JBZ_005	F	1961	59	-	-
JBZ_006	M	1953	67	-	-
JBZ_007	M	1947	73	-	-
JBZ_008	F	1952	69	-	-
JBZ_009	M	1948	73	-	-
JBZ_010	M	1948	73	-	-
JBZ_011	F	1949	72	-	-
JBZ_012	F	1944	77	-	-
JBZ_013	F	1960	61	-	-
JBZ_014	M	1936	85	-	-
JBZ_015	M	1953	68	-	-
JBZ_016	M	1949	72	-	-
JBZ_017	F	1968	54	-	-
JBZ_018	F	1952	70	-	-
JBZ_019	F	1948	74	-	-
JBZ_020	M	1940	82	-	-
JBZ_021	F	1942	80	-	-
JBZ_022	M	1958	61	-	-
JBZ_023	M	1964	55		-
JBZ_024	F	1949	69	-	-
JBZ_025	M	1940	78	-	-
JBZ_026	M	1942	76	-	-
JBZ_027	M	1957	59	-	-
JBZ_028	M	1949	68	-	-
JBZ_029	F	1942	74	-	-
JBZ_030	F	1955	61	-	-
JBZ_031	F	1956	60	-	-
JBZ_032	M	1951	60	-	-
JBZ_033	M	1934	80	-	-
*ADNI*					
005_S_0448	M	1920	90	3,4	2
005_S_4910	F	1931	81	2,3	4
006_S_4546	M	1941	71	3,8	2
011_S_0008	F	1921	85	9,4	3
011_S_0010	F	1931	76	11	2
011_S_0053	M	1925	82	3,8	3
011_S_0183	F	1933	74	2,4	3
011_S_1282	F	1930	81	0,8	4
011_S_4366	M	1938	73	4,3	4
011_S_4827	M	1941	76	1,8	5
011_S_4845	F	1944	68	8,9	2
014_S_0563	M	1925	85	7,9	2
014_S_4039	M	1955	56	2,9	4
014_S_4263	M	1937	76	1,5	2
016_S_0702	M	1921	92	1	2
023_S_0042	M	1932	80	0,9	1
024_S_0985	M	1925	87	1,2	4
024_S_4223	M	1935	75	0,1	3
027_S_0408	M	1924	89	1,6	2
027_S_4802	M	1929	83	0,6	3
027_S_4936	M	1934	78	2,8	3
027_S_4962	F	1932	80	5,8	3
029_S_1384	M	1935	73	4,8	3
031_S_0830	M	1935	75	1	1
032_S_0214	M	1941	72	3,5	4
032_S_2119	M	1939	78	0,5	1
037_S_6216	M	1934	84	2	4
073_S_0565	M	1931	75	1,2	3
082_S_5029	M	1933	79	4,3	3
114_S_1118	M	1924	89	1,3	2
114_S_5234	F	1934	83	4,8	4
116_S_0834	M	1942	68	0	3
116_S_6100	F	1946	71	1,4	3
123_S_4526	M	1932	81	1,4	3
123_S_6891	M	1942	78	0,6	4
126_S_0709	M	1922	91	2,9	2
126_S_4675	M	1932	81	2,1	1
127_S_4240	M	1940	72	5,1	2
127_S_4765	M	1936	81	0,1	3
127_S_4928	M	1934	82	4,2	4
127_S_5058	M	1951	61	4,1	3
127_S_5218	F	1930	82	1,3	5
129_S_4422	F	1941	76	4,9	1
*HC*					
JBZ_034	F	1946	74	-	-
JBZ_035	M	1960	61	-	-
JBZ_036	M	1952	68	-	-
JBZ_037	M	1947	73	-	-
JBZ_038	F	1953	67	-	-
JBZ_039	M	1944	76	-	-
JBZ_040	M	1942	77	-	-
JBZ_041	F	1976	44	-	-
JBZ_042	M	1965	55	-	-
JBZ_043	F	1961	59	-	-
JBZ_044	F	1950	70	-	-
JBZ_045	M	1972	48	-	-
JBZ_046	F	1959	60	-	-
JBZ_047	F	1959	60	-	-
JBZ_048	M	1955	64	-	-
JBZ_049	M	1964	54	-	-
JBZ_050	F	1957	61	-	-
JBZ_051	F	1955	58	-	-
JBZ_052	M	1951	65	-	-

Participant demographics are shown. Disease Stage at Autopsy is classified according to the modified McKeith criteria (3): (1) Brainstem predominant; (2) Limbic (transitional); (3) Neocortical (diffuse); (4) Amygdala predominant; (5) Olfactory bulb

- = Not Available

### Statistical parametric mapping

Statistical parametric mapping (SPM) analyses were performed with SPM12 (Wellcome Department of Imaging Neuroscience Group, London, UK; http://www.fil.ion.ucl.ac.uk/spm). This software was also employed for further post-processing of the data.

### Image processing

Following reorientation of brain images to ensure alignment with the anterior commissure, inter-subject alignment was achieved utilizing a discrete cosine transform model. Spatial transformation to MNI space, combined with warping of the individual subject to a standardized template, was facilitated by the normalization module available in SPM12. Specifically, the Dementia-Specific [^18^F]FDG PET template was used for this purpose [20]. Image modulation was omitted to conserve diagnostic accuracy in the post-processed data [21]. Finally, a standard 8-mm FWHM Gaussian kernel smoothing was applied to increase the signal-to-noise ratio [22].

### Statistical analysis

Statistical Parametric Mapping was performed using a generalized linear model with an independent two-sample *t*-test design. At each voxel, the dependent variable was normalized [18F]FDG uptake, and the independent variable was group membership (DLB versus healthy controls). No additional covariates were included in the primary model. Hypermetabolism was defined as significantly higher normalized [18F]FDG uptake in patients than in healthy controls. Statistical significance was assessed voxel-wise across the whole brain using family-wise error (FWE) correction at *P* < 0.01, to account for multiple comparisons. This threshold was chosen to minimize false-positive findings and to maximize confidence in the identified hypermetabolic pattern, in line with the aim of establishing disease-specific pathophysiology and potential diagnostic biomarkers. The analysis was conducted at the whole-brain level rather than within predefined regions of interest. Subregional uptake values were therefore not extracted a priori, and significant clusters were anatomically localized only after voxel-wise analysis.

For thresholding, a standard relative threshold of 0.8 was applied, excluding voxels with values below 80% of the subject’s mean global uptake. Grand mean scaling was set to the default value of 50, providing an intuitive scale without affecting the statistical analysis [23]. Proportional normalization was performed relative to the whole-brain mean voxel value. In addition, the brain mask applied in the generalized linear model was derived from the FieldMap toolbox in SPM12 [20]. To reduce the likelihood of small spurious findings, a minimum cluster extent threshold of 20 voxels was applied. Results were visualized with the xjView toolbox (https://www.alivelearn.net/xjview) and MRIcroGL [24].

To validate the findings from our patient cohort, we replicated the analysis using the ADNI cohort in comparison with our healthy controls. Within the ADNI cohort, an additional analysis was performed to control for disease stage. The same pipeline described above was used, with disease stage at autopsy included as a covariate, resulting in a group-level ANCOVA controlling for disease stage.

## Results

In total, 33 patients (14 females; 42%) with DLB were included from the prospectively kept database at Jeroen Bosch Hospital (The Netherlands). The mean age of the included patients was 69.2 years (± 7.7 years). The age and gender-matched controls with a normal brain metabolism (9 females; 45%) had a mean age of 62.2 ± 9.4 years.

The ADNI validation cohort consisted of 43 patients (11 females; 26%) with DLB. The mean age of this cohort was 78.8 years (± 7.5 years). The mean age difference between the latest [^18^F]FDG PET scan and the date of autopsy was 3.0 years (± 2.6 years). The mean disease stage was 2.8 (± 1.1).

SPM analysis revealed a wide range of brain regions exhibiting metabolic dysfunction involved in dementia with Lewy bodies. A detailed report of each anatomical region is provided. The scope of this study does not encompass cortical metabolism, which is therefore referenced solely in the description of the figures. Regions of significant differences in metabolism were analyzed using conjunction analysis. Results shown were FWE-corrected at *P* < 0.01.

### Comparison of DLB patients with healthy controls

Increased metabolism was seen in the bilateral centrum semiovale; bilateral putamen; bilateral globus pallidus; bilateral anterior and posterior limb of capsula interna; left nucleus accumbens; bilateral genu of the corpus callosum; bilateral ventral lateral nucleus of the thalamus; bilateral ventral anterior nucleus of the thalamus; bilateral ventral posterolateral nucleus of the thalamus.

Within the brainstem, three areas of increased metabolism were identified. These being the bilateral posterior pons; bilateral tectum mesencephali; bilateral superior cerebellar peduncle (SCP). Cerebellar regions possessing increased metabolism were the bilateral deep cerebellar nuclei; bilateral vermis of the cerebellum; bilateral declive of the cerebellum.

No regions with hypometabolism in subcortical, brainstem or cerebellar regions were observed when comparing patients with DLB to the controls.

**Fig. 1:**
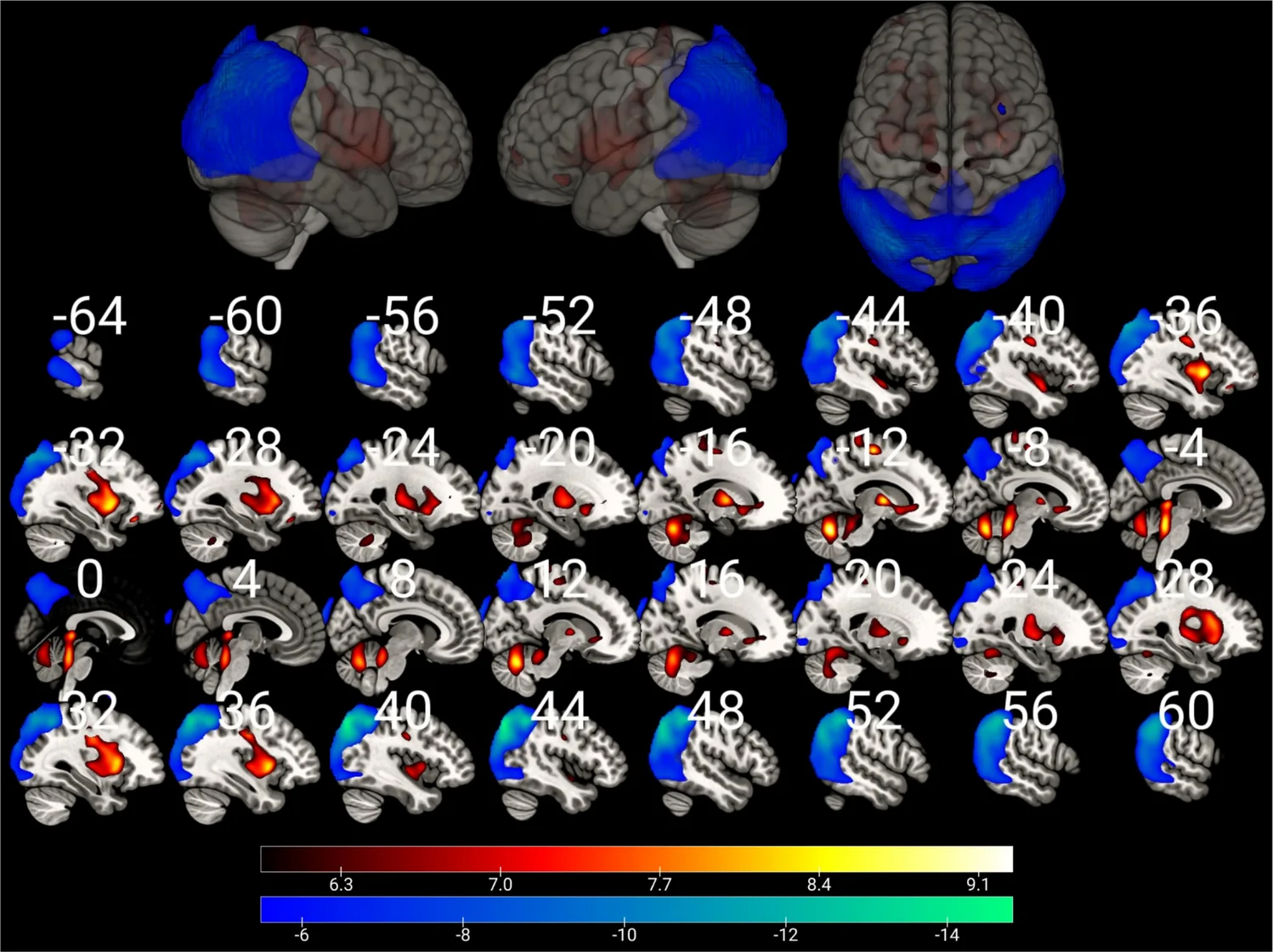
Regional [^18^F]FDG PET alterations in glucose metabolism in patients with DLB (JBZ cohort) when compared to healthy controls. Changes in metabolism within subcortical structures can be appreciated in the bilateral internal capsule of the deep white matter. Also the thalamic formation, putamen, and globus pallidus of the basal ganglia system, the tectum mesencephali, superior cerebellar peduncle, dorsal pons in the brainstem, and the deep cerebellar nuclei. All subcortical structures exclusively exhibit altered hypermetabolism. Regarding cortical regions, hypermetabolism is seen in the right anterior cingulate gyrus; bilateral orbitofrontal cortex; bilateral anterior superior frontal gyrus; bilateral insula; bilateral paracentral lobule; left superior temporal gyrus. Cortical hypometabolism was discovered in the bilateral precuneus; bilateral superior parietal lobe; bilateral superior occipital gyrus; bilateral middle occipital gyrus; bilateral inferior occipital gyrus; bilateral supramarginal gyrus; bilateral angular gyrus; bilateral posterior inferior temporal gyrus; bilateral posterior middle temporal gyrus; right superior temporal gyrus. Regions of significant differences in hypometabolism and hypermetabolism are displayed on blue–green and red–white scales, respectively, with darker blue indicating greater hypometabolism and brighter white indicating greater hypermetabolism. DLB = Dementia with Lewy bodies; FDG = [^18^F]Fluorodeoxyglucose; FWE = Family Wise Error

The aforementioned differences in brain glucose metabolism between healthy subjects and subjects suffering from DLB can be observed in Fig. 1**.**

The aforementioned results were replicated in the ADNI cohort. More particularly, increased metabolism was seen in the bilateral putamen; bilateral globus pallidus; bilateral pulvinar; bilateral ventral posterolateral nucleus; bilateral ventral lateral nucleus; bilateral splenium of corpus callosum.

Within the brainstem, the three regions that showed heightened metabolism are the bilateral tectum mesencephali; bilateral SCP; bilateral posterior pons.

Cerebellar regions consisted of the bilateral deep cerebellar nuclei; bilateral culmen of the cerebellum; bilateral vermis of the cerebellum; bilateral tonsil of the cerebellum; bilateral semi-lunar lobe of the cerebellum.

Subcortical hypometabolism was present in the bilateral splenium of the corpus callosum; bilateral anterior thalamic complex; bilateral head of the caudate nucleus; bilateral hippocampus; corpus of right caudate nucleus; bilateral mediodorsal nucleus of the thalamus; bilateral anteroventral nucleus of the thalamus; right anteromedial nucleus of the thalamus.

**Fig. 2:**
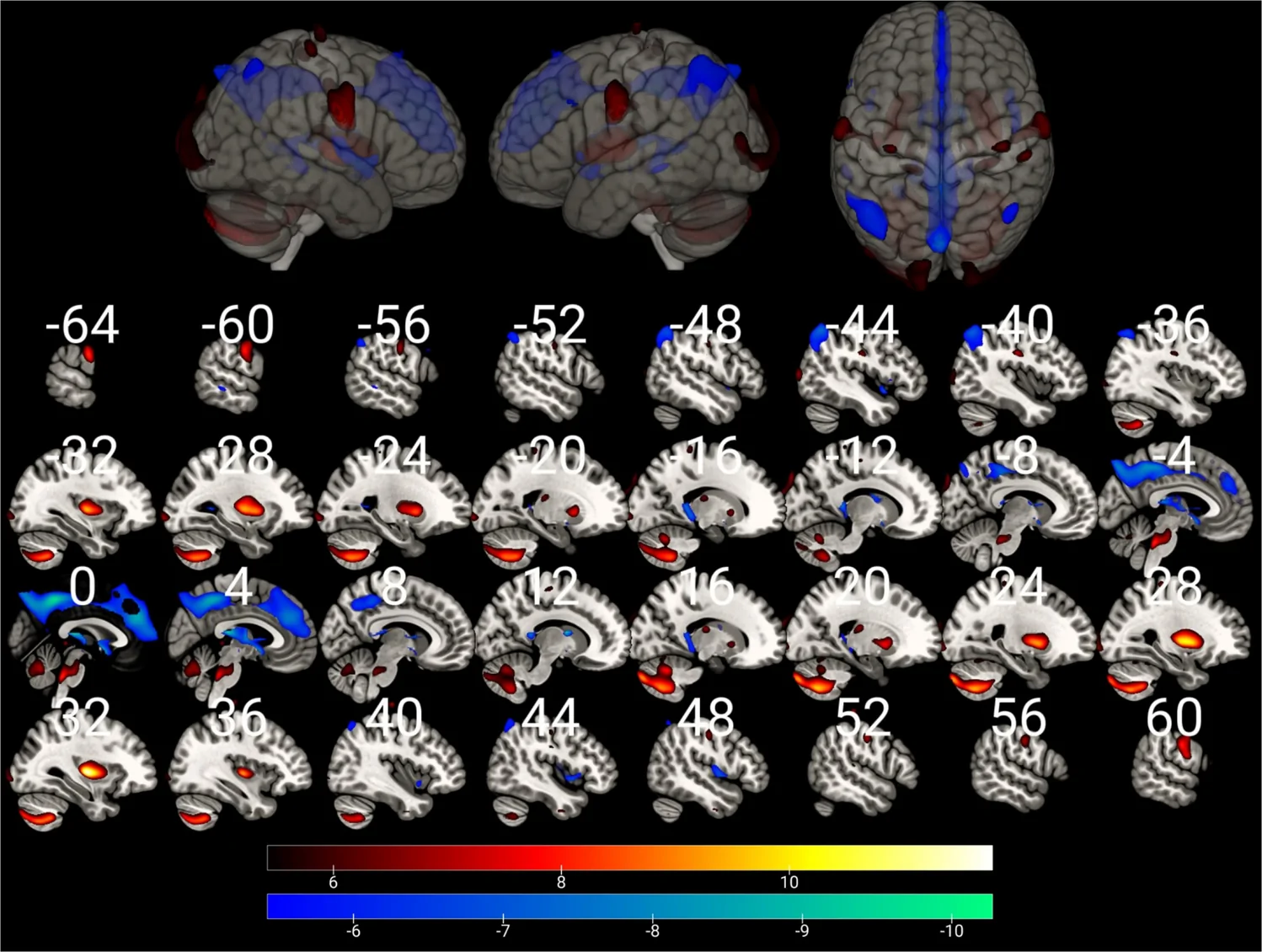
Regional [^18^F]FDG PET alterations in glucose metabolism in patients with DLB (ADNI cohort) when compared to healthy controls. Increases in metabolism within subcortical structures can be appreciated in the splenium of corpus callosum. Also the thalamic formation, putamen, and globus pallidus of the basal ganglia system, the tectum mesencephali, superior cerebellar peduncle, dorsal pons in the brainstem. Cerebellar regions consist of deep cerebellar nuclei, culmen, vermis, tonsil, and semi-lunar. Regarding cortical region, hypermetabolism is seen in the right inferior temporal gyrus; bilateral paracentral lobule; bilateral postcentral gyrus (hand + face area); bilateral inferior occipital gyrus; bilateral middle occipital gyrus; bilateral superior occipital gyrus. Subcortical hypometabolism is present in the bilateral splenium of corpus callosum; anterior and medial thalamic complexes; head of caudate nucleus; corpus of right caudate nucleus; hippocampus. Within the brainstem reduced metabolism is seen in the bilateral zona incerta. Cortical regions with observed hypometabolism are left middle temporal gyrus; bilateral superior frontal gyrus; bilateral supplemental motor area; bilateral angular gyrus; bilateral precuneus; bilateral anterior cingulate gyrus; bilateral middle cingulate gyrus; bilateral superior parietal lobule; bilateral insula; bilateral olfactory cortex. Regions of significant differences in hypometabolism and hypermetabolism are displayed on blue–green and red–white scales, respectively, with darker blue indicating greater hypometabolism and brighter white indicating greater hypermetabolism. DLB = Dementia with Lewy bodies; FDG = [^18^F]Fluorodeoxyglucose

Within the brainstem, reduced metabolism was seen in the bilateral zona incerta, as can be seen in Fig. 2.

### Covariate analysis adjusting for disease stage

After adjusting for disease stage by introducing a covariate, hypermetabolism in non-cortical regions was observed only in the bilateral putamen.

Subcortical hypometabolism was present in the bilateral splenium of corpus callosum; corpus of right caudate nucleus; right mediodorsal nucleus of the thalamus; right anteroventral nucleus of the thalamus.

**Fig. 3:**
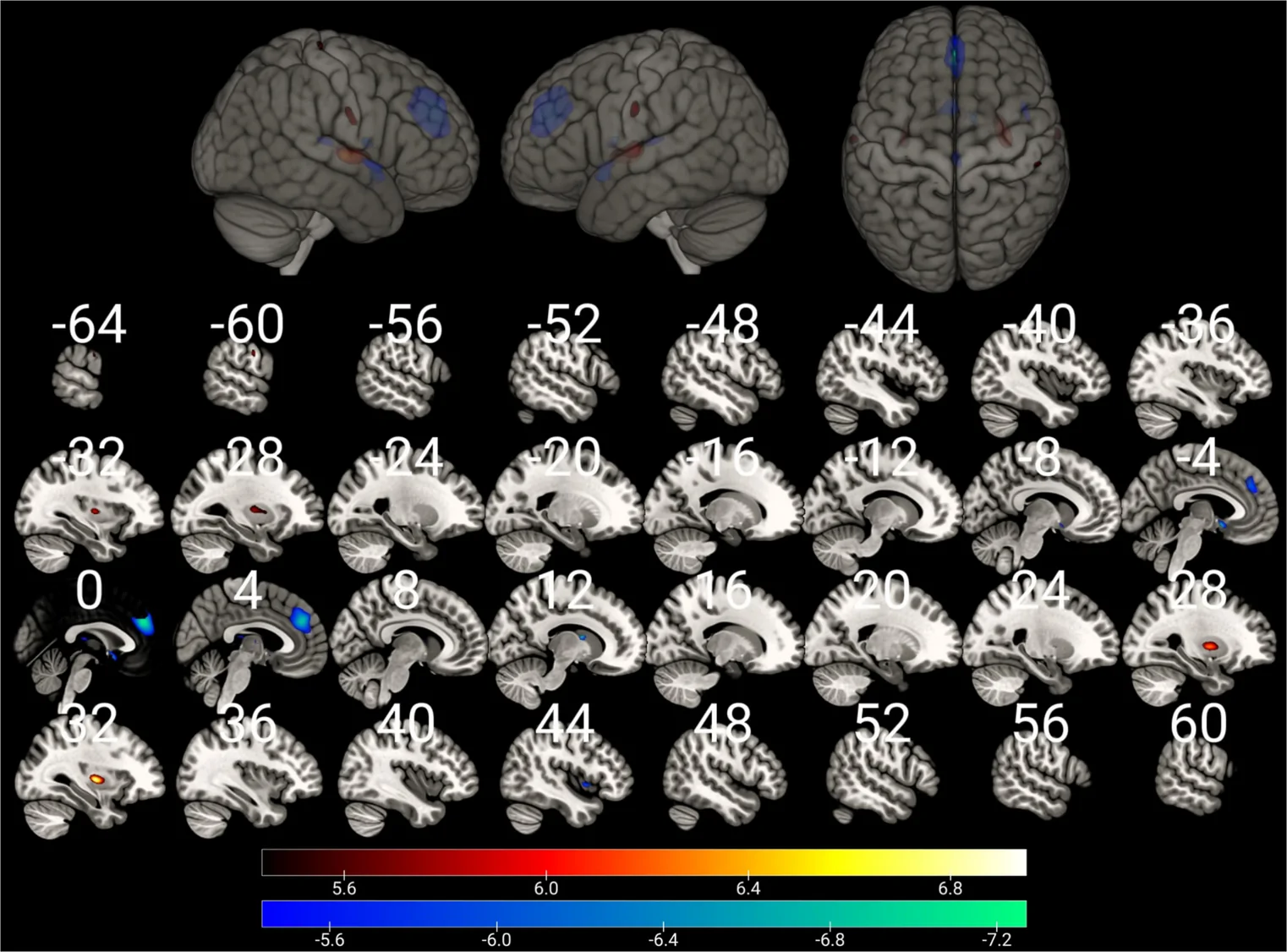
Regional [^18^F]FDG PET alterations in glucose metabolism in patients with DLB (ADNI cohort) when compared to healthy controls, corrected for disease stage at autopsy. Hypermetabolism in non-cortical regions is observed in the bilateral putamen. Subcortical hypometabolism is present in the bilateral splenium of corpus callosum; corpus of right caudate nucleus; right medial thalamic complex. Regarding cortical regions, hypermetabolism is seen in the hand area of the bilateral postcentral gyrus. Hypometabolism is seen in the right insula; left olfactory cortex; bilateral superior frontal gyrus; bilateral anterior cingulate cortex. Regions of significant differences in hypometabolism and hypermetabolism are displayed on blue–green and red–white scales, respectively, with darker blue indicating greater hypometabolism and brighter white indicating greater hypermetabolism. DLB = Dementia with Lewy bodies; FDG = [^18^F]Fluorodeoxyglucose

Changes in brain glucose metabolism were illustrated in Fig. 3.

Changes in brain glucose metabolism for all three analyses are described in Table 2.

**Table 2:** cluster results of Statistical Parametric Mapping (SPM) analyses

X	Y	Z	Cluster volume (mm^3^)	*T*-value	*P*-value	Metabolism	Anatomical region
*JBZ vs HC*							
−12	−64	−26	6562	9.25	0.000	Hyper	Deep cerebellar nuclei_L
−6	−40	−20		8.82	0.000	Hyper	Dorsal pons_L/R, superior cerebellar peduncle_L/R, tectum mesencephali_L/R
12	−64	−28		8.67	0.000	Hyper	Deep cerebellar nuclei_R
−34	4	0	4487	8.97	0.000	Hyper	Putamen_L, globus pallidus_L, Internal capsule_L, superior temporal gyrus_L
−12	−6	6		8.61	0.000	Hyper	Thalamic formation_L
−30	12	6		8.09	0.000	Hyper	Insula_L
−12	−16	60	609	8.61	0.000	Hyper	Paracentral lobule_L
−10	−32	70		6.99	0.000	Hyper	Paracentral lobule_L
34	10	4	3952	8.47	0.000	Hyper	Thalamic formation_R, putamen_R, globus pallidus_R
34	−16	36		7.90	0.000	Hyper	Internal capsule_R
36	−12	2		7.42	0.000	Hyper	Insula_R
−36	30	−16	153	8.05	0.000	Hyper	Orbitofrontal cortex_L
−46	28	−12		6.63	0.000	Hyper	Orbitofrontal cortex_L
−32	60	−2	101	7.38	0.000	Hyper	Anterior superior frontal gyrus_L
−28	64	4		6.85	0.000	Hyper	Anterior superior frontal gyrus_L
−40	54	−4		5.82	0.004	Hyper	Orbitofrontal cortex_L
22	−28	60	482	6.78	0.000	Hyper	Paracentral lobule_R
12	−20	60		6.75	0.000	Hyper	Anterior cingulate gyrus_R
14	−30	72		6.65	0.000	Hyper	Paracentral lobule_R
26	62	−6	102	6.75	0.000	Hyper	Anterior superior frontal gyrus_R
26	46	−16		6.22	0.001	Hyper	Orbitofrontal cortex_R
42	−78	48	29463	−14.81	0.000	Hypo	Superior occipital gyrus_R, middle occipital gyrus_R, inferior occipital gyrus_R
44	−54	52		−13.29	0.000	Hypo	Precuneus_R, superior parietal lobe_R, supramarginal gyrus_R, angular gyrus_R, posterior inferior temporal gyrus_R, posterior middle temporal gyrus_R, superior temporal gyrus_R
−36	−82	52		−11.66	0.000	Hypo	Precuneus_L, superior parietal lobe_L, superior occipital gyrus_L, middle occipital gyrus_L, inferior occipital gyrus_L, supramarginal gyrus_L, angular gyrus_L, posterior inferior temporal gyrus_L, posterior middle temporal gyrus_L
*ADNI vs HC*							
32	−12	4	1015	11.78	0.000	Hyper	Putamen, globus pallidus_R
64	−12	4	585	10.15	0.000	Hyper	Postcentral gyrus – face area_R
52	−12	40		7.01	0.000	Hyper	Postcentral gyrus – face area_R
−30	−12	4	801	10.11	0.000	Hyper	Putamen, globus pallidus_L
−28	2	2		9.15	0.000	Hyper	Putamen, globus pallidus_L
20	−66	−48	2888	9.84	0.000	Hyper	Vermis, tonsil, semi-lunar lobule, and deep cerebellar nuclei_R
18	−86	−38		9.64	0.000	Hyper	Semi-lunar lobule_R
34	−54	−48		8.90	0.000	Hyper	Culmen_R
−24	−64	−48	1654	9.02	0.000	Hyper	Vermis, tonsil, and semi-lunar lobule_L
−30	−58	−48		8.90	0.000	Hyper	Culmen_L
−14	−64	−28		7.50	0.000	Hyper	Deep cerebellar nuclei_L
−62	−10	32	394	8.58	0.000	Hyper	Postcentral gyrus – face area_L
−48	−14	42		6.26	0.001	Hyper	Postcentral gyrus – face area_L
−2	−38	−34	619	8.37	0.000	Hyper	Dorsal pons_L/R, superior cerebellar peduncle_L/R
−4	−26	−28		7.05	0.000	Hyper	Dorsal pons_L/R, tectum mesencephali_L/R, superior cerebellar peduncle_L/R
22	−102	−2	598	7.30	0.000	Hyper	Middle occipital gyrus_R
22	−104	20		6.30	0.001	Hyper	Superior occipital gyrus_R
42	−88	−2		5.57	0.006	Hyper	Inferior occipital gyrus_R
−40	−18	32	85	7.16	0.000	Hyper	Postcentral gyrus – face area_L
54	−26	68	49	7.13	0.000	Hyper	Postcentral gyrus – hand area_R
38	−20	78	55	7.05	0.000	Hyper	Paracentral lobule_R
18	−18	18	86	7.03	0.000	Hyper	Thalamic formation_R
−16	−104	−2	803	6.98	0.000	Hyper	Inferior occipital gyrus_L, middle occipital gyrus_L
−28	−98	−6		6.97	0.000	Hyper	Middle occipital gyrus_L
−10	−94	40		6.74	0.000	Hyper	Superior occipital gyrus_L
2	−36	0	27	6.83	0.000	Hyper	Splenium of corpus callosum_L + R
44	−4	−40	47	6.61	0.000	Hyper	Inferior temporal gyrus_R
36	−4	−42		6.14	0.001	Hyper	Inferior temporal gyrus_R
−18	−22	18	63	6.60	0.000	Hyper	Thalamic formation_L
−32	−22	82	28	6.58	0.000	Hyper	Paracentral lobule_L
32	−30	64	68	6.58	0.000	Hyper	Postcentral gyrus – face area_R
−18	−34	68	59	6.22	0.001	Hyper	Postcentral gyrus – face area_L
2	−28	14	1276	−10.27	0.000	Hypo	Splenium of corpus callosum_L/R, hippocampus_L/R, zona incerta_L/R
12	0	14		−9.45	0.000	Hypo	Anterior and medial thalamic complexes_L/R, head of caudate nucleus_L/R, corpus of caudate nucleus_R
2	6	−8		−9.33	0.000	Hypo	Olfactory cortex_L/R
0	−48	48	4264	−9.74	0.000	Hypo	Precuneus_L/R, superior parietal lobule_L/R, middle cingulate gyrus_L/R
0	42	30		−8.70	0.000	Hypo	Superior frontal gyrus_L/R, supplemental motor area_L/R, anterior cingulate gyrus_L/R, middle cingulate gyrus_L/R
0	−72	44		−8.20	0.000	Hypo	Precuneus_L/R
−26	−50	8	48	−7.77	0.000	Hypo	Precuneus_L
−44	−62	56	780	−7.42	0.000	Hypo	Angular gyrus_L
−54	−54	46		−6.20	0.001	Hypo	Angular gyrus_l
−42	−68	40		−6.09	0.001	Hypo	Angular gyrus_L
46	4	−4	203	−7.16	0.000	Hypo	Insula_R
−44	8	−8	71	−6.87	0.000	Hypo	Insula_L
−44	12	0		−6.06	0.001	Hypo	Insula_L
44	−60	58	109	−6.09	0.001	Hypo	Angular gyrus_R
−60	−34	−8	36	−5.80	0.003	Hypo	Middle temporal gyrus_L
−54	−44	−2		−5.45	0.009	Hypo	Middle temporal gyrus_L
*ADNI vs HC – corrected*							
32	−10	2	140	6.95	0.000	Hyper	Putamen_R
−30	−12	6	76	6.20	0.001	Hyper	Putamen_L
64	−6	24	22	5.95	0.002	Hyper	Postcentral gyrus – hand area_R
−62	−10	30	23	5.91	0.002	Hyper	Postcentral gyrus – hand area_L
2	42	30	522	−7.27	0.000	Hypo	Superior frontal gyrus_L/R, anterior cingulate cortex_L/R
−4	6	−10	80	−7.07	0.000	Hypo	Olfactory cortex_L
10	2	10	29	−6.69	0.000	Hypo	Caudate nucleus_R
2	−26	14	54	−6.10	0.001	Hypo	Splenium of corpus callosum_L/R
4	−8	8		−5.96	0.002	Hypo	Medial thalamic complex_R

Peak *T*-value coordinates are provided for each cluster, together with cluster volumes and associated anatomical regions. *P*-values are peak-level corrected for Family-Wise Error (FWE)

mm^3^ = cubic millimeter; Hyper = hypermetabolism; Hypo = hypometabolism

## Discussion

In this study, we show that individuals with DLB exhibit widespread hypermetabolism of subcortical grey matter in comparison to healthy individuals. The most notable findings include the anterolateral thalamus, deep cerebellar nuclei, and mesencephalic tectum.

Glucose metabolism is a main diagnostic marker in differentiating between dementia causes. Specific patterns are therefore well-studied in dementia neuroimaging, and this is also true for Dementia with Lewy bodies. Characteristic metabolic changes in DLB are hypometabolism in the occipital, parietal, and temporal lobes [10]. Additionally, relatively spared posterior cingulate metabolism is observed with hypometabolism in the precuneus, forming the cingulate island sign [25]. Hypermetabolism, on the other hand, has been repeatedly shown in the orbitofrontal cortex, pallidum, putamen, central cerebellum, somatomotor cortices, and pons, with a few studies also reporting the insula or anterior cingulate cortex [26–29]. These regions are validated by our study. Our study re-establishes the cortical metabolic signatures of DLB, including the cingulate island sign, the occipital tunnel sign, and the occipital pole hypometabolism sign [30, 31], which further validates our results.

By contrast, the subcortical findings are less well established. Interestingly, consensus on the metabolism of the thalamus in patients with DLB has not yet been reached [27, 28]. On the other hand, hypermetabolism relative to the global mean intensity of the brain are reported features of DLB in the bilateral amygdala, hippocampi, and parahippocampal gyri, which were not seen in our cohort. In the validation cohort, hypermetabolism emerged in the hippocampi only [28, 32]. To our knowledge, metabolism of the mesencephalic tectum and superior cerebellar peduncle has not been explicitly described to date and therefore represents the main novel finding of the present study.

The hypermetabolic pattern of the SCP and mesencephalic tectum does not appear to be specific to patients showing a cingulate island sign, making it a potentially promising diagnostic marker. While our own cohort demonstrates a cingulate island sign, the ADNI cohort does not. However, both cohorts show the same hypermetabolism in the SCP and mesencephalic tectum. This finding may prove valuable in aiding diagnosis. Approximately 50% of all patients with DLB also present with concomitant amyloid pathology, which eliminates the cingulate island sign. For this reason, the cingulate island sign has limited sensitivity for DLB diagnosis. These initial findings may provide a biomarker with markedly increased sensitivity. However, formal sensitivity and specificity analyses should be conducted in the future.

After controlling for disease stage, the previously observed hypermetabolic pattern in the SCP and mesencephalic tectum was no longer significant. Disease stages characterized by α-synuclein accumulation in the amygdala or olfactory bulb rather than in brainstem regions did not alter this result. Together, these findings suggest that the observed activity is closely related to disease progression rather than representing a stage-independent feature. However, this interpretation should be made cautiously, as disease stage may itself reflect multiple factors, including disease duration, clinical severity, and α-synuclein burden. Alternatively, progressive basal ganglia involvement, a hallmark feature of DLB, may drive SCP metabolism. This could help explain the persistence of this pattern across progression stages. Whether such changes represent a compensatory adaptation or a pathological process remains to be determined.

### Basal ganglia metabolism of Lewy body dementia; new insights into an established framework

DLB has long been recognized to cause dopaminergic dysfunction in the basal ganglia, disrupting functional connectivity and metabolism [33, 34]. This established pattern is also presented in our data, showcasing an increased metabolic signature in the putamen and globus pallidus [28, 35, 36]. Metabolic increases in the basal ganglia of patients with DLB are shown to correlate with the degree of nigrostriatal dopaminergic depletion [37]. Putaminal hypermetabolism has therefore been repeatedly investigated as a supportive imaging biomarker for distinguishing DLB from Alzheimer’s disease [27, 38, 39]. The basal ganglia network is an extensively studied functional unit, responsible for, among other things, movement disorders such as parkinsonism caused by nigrostriatal dopaminergic depletion as seen in DLB [40]. Recently, this network was expanded upon when evidence of a disynaptic projection from the subthalamic nucleus (STN) directly to the cerebellar cortex emerged. This was described by the injection of a retrograde transneuronal traveling virus into multiple regions of the cerebellar cortex. These studies found connections originating in the STN, first innervating the pontine nuclei and then second-order neurons innervating the cerebellar cortex [41, 42]. A functional disynaptic connection from the dentate nucleus to the striatum was identified in a similar way. This bidirectional mode of communication gave rise to the concept of an integrated functional cerebellar-basal ganglia network [43]. New insights into the potential for a more important cerebellar role in parkinsonian symptoms were formed on this basis, although the function of this cerebellar role (whether compensatory of pathological) is not yet understood.

Moreover, STN hyperactivation is an extensively studied subject in parkinsonian disease pathology. Given that the STN projects directly to the cerebellar cortex, using glutamate as a neurotransmitter, cerebellar cortex hyperactivity is anticipated [44]. Consequently, this is expected to activate the nucleus dentatus of the cerebellum and the dentatorubrothalamic tract through its excitatory connections. This potential mechanism may explain the increased activity observed in cerebellar regions and the SCP in our study.

### Hypermetabolism in the dentate nucleus and the anterolateral thalamus complex: a role for the superior cerebellar peduncle?

**Fig. 4:**
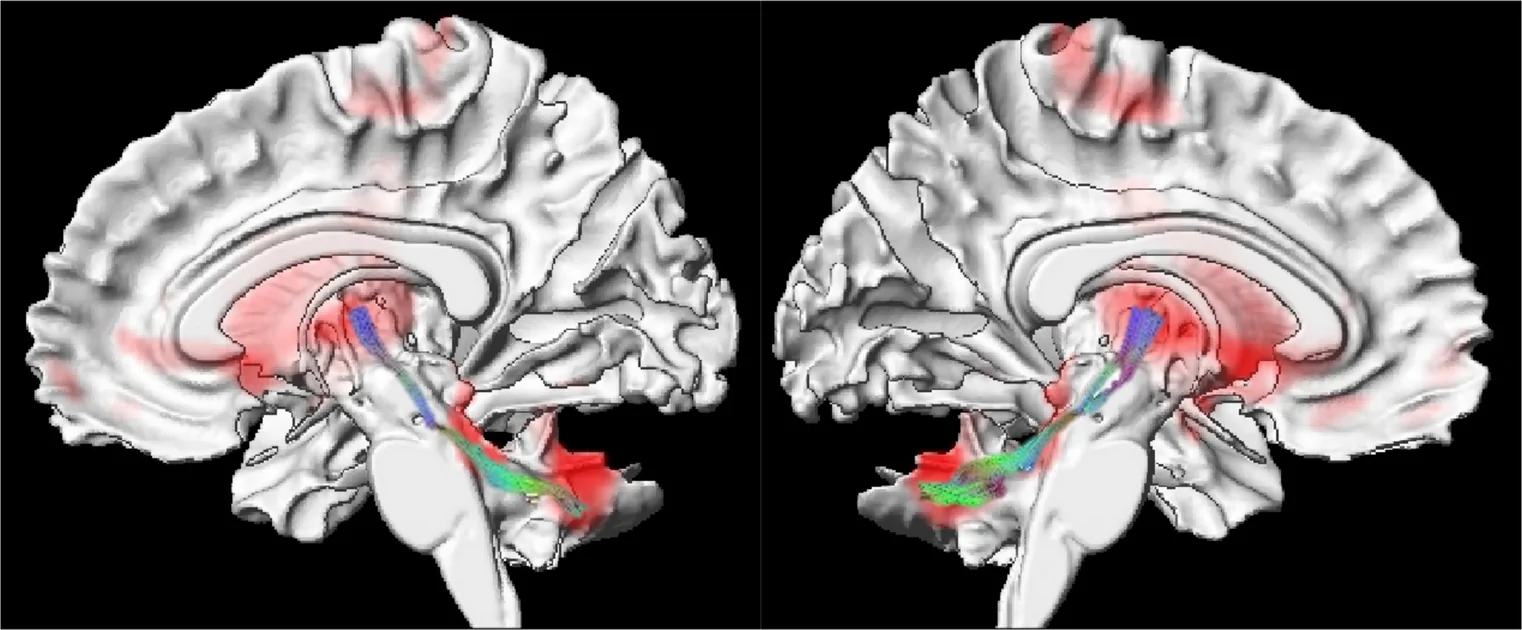
Regional [^18^F]FDG PET hypermetabolism in patients with DLB (JBZ cohort) when compared to healthy controls, sagittal midline view. Dentatorubrothalamic tract (DRTT) was delineated from human connectome data with a seed region placed in the dentate nucleus (Julich-Brain Atlas) and region-of-interests in the contralateral red nucleus (BrainSeg atlas) and thalamus (BrainSeg atlas) [65–67]. Regions of significant differences in hypermetabolism are shown in red, with tractography of the bilateral DRTT overlaid for contextual reference. DLB = Dementia with Lewy bodies; FDG = [^18^F]Fluorodeoxyglucose

We observed significantly increased metabolism in the dentate nucleus and anterolateral thalamic nuclei. These nuclei mark initiation and termination points of the dentatorubrothalamic tract, as shown in Fig. 4.

The SCP serves as the primary outflow tract of the cerebellum and comprises efferent fibers originating from the globose nucleus, emboliform nucleus, and dentate nucleus. More than 75% of all efferent fibers from the cerebellum originate from the dentate nucleus, creating the dentatorubrothalamic tract, signifying the importance of this nucleus and its tracts in the transmission of cerebellar signals [45]. We did not observe altered activity in the nucleus ruber, which can be explained by the fact that a substantial portion of the fibers in this tract bypass the nucleus ruber on their course to the thalamus [46].

Previous anatomical and imaging studies have demonstrated that the decussating pathway of the dentato-rubrothalamic tract (DRTT) primarily projects to anterior and lateral thalamic nuclei. Conversely, posterior and medial thalamic nuclei account for projection sites for nondecussating fibers [47]. These findings have raised questions about fuctional differences between these pathways. Interestingly, a delineation of activity within the thalamic complex is apparent in our data. Hypermetabolism was observed predominantly within the lateral and ventral nuclei, aligning with the trajectory of the decussating DRTT [47]. This would suggest that cerebellar-mediated activity of the thalamus in patients with DLB is primarily routed through the decussating pathway of the DRTT, specifically. However, how these findings correlate with clinical symptoms, remains elusive and warrants further research.

Primarily known for relaying cerebellar motor functions, the dentato-rubro-thalamic tract (DRTT) also contributes to cognition through its prefrontal and parietal connections [48]. In patients with Huntington’s disease, microstructural integrity of the DRTT has been correlated with both motor and cognitive impairments [49]. Similarly, in Parkinson’s disease, hypermetabolism has been observed in regions comparable to those identified in our DLB cohort, particularly within the cerebellum and brainstem. Although thalamic changes were not significant at strict thresholds, more liberal thresholds additionally revealed hypermetabolism in the prefrontal cortex and dentate nucleus [50], consistent with the trajectory of DRTT connectivity.

### Novel insights into the metabolism of the mesencephalic tectum in patients with dementia with Lewy bodies

Distinct hypermetabolism in the midbrain tectum is noted as well. Traditionally, the superior colliculus is associated with its role in physically redirecting sensory structures of the head toward stimuli of interest by orienting eye, head, and arm movement and primarily generating eye saccadic movements [51]. However, while this holds true for the superficial cell layers, the deep layers serve as a multimodal sensory processing and attention node, integrating visual, auditory, and sensorimotor information [52]. Moreover, these deep layers receive GABAergic projections from the substantia nigra pars reticularis, which, in turn, is receives projections from the caudate nucleus and putamen. Most likely, also through inhibitory GABA neurons [53]. Our data demonstrate increased metabolism in the putamen. This heightened activity potentially induces increased inhibition in the substantia nigra pars reticularis. Through this pathway, disinhibition of the superior colliculus occurs, resulting in the hypermetabolism observed in the tectum.

Additionally, the cerebellum projects to the superior colliculus via two pathways. Firstly, the dentate nucleus and posterior interposed nucleus connect contralaterally to this nucleus in macaque monkeys [54]. Both of these deep cerebellar nuclei are glutamatergic [55]. Secondly, the dorsal fastigial nucleus sends bilateral projections [54]. This part of the nucleus also employs glutamate, stimulating the targeted superior colliculus [56]. These cerebellar efferents provide further mechanistic insights into the increased activity observed in the superior colliculus.

### Limitations and future research

The innovative approach of this study to focus on subcortical structures, combined with the multi-cohort approach, the application of statistical parametric mapping, and the use of family-wise error correction contributes to the robustness of the results, minimizing the likelihood of false positives. However, the limited sample size, particularly within the autopsy subcohorts, and the retrospective study design constitute limitations of this study. In addition, no direct quantitative measures of α-synuclein burden in the McKeith-staging regions were available.

The nature of apparent hypermetabolism in SPM analyses remains debated, with some suggesting it could be a normalization artefact [57]. Proportional normalization of each individual within the analysis measures hypermetabolism relative to whole-brain mean glucose metabolism, not as an absolute value. Thus, hypermetabolism may be due to relative disease-sparing within a region, even if absolute metabolism remains lower than in controls. However, in regions showing hypermetabolism on SPM analysis, absolute FDG uptake quantification with arterial sampling reveal true hypermetabolism in subcortical and infratentorial regions of patients with Parkinson’s disease. This supports that apparent hypermetabolism signifies true hypermetabolism [58]. Furthermore, similar findings in other α-synucleinopathies support the interpretation of these results as reflecting true hypermetabolism [50]. If the observed hypermetabolic pattern were attributable to a normalization artefact, a similar pattern would be expected in other neurodegenerative dementias characterized by widespread cortical hypometabolism. Direct comparison with the existing literature is challenging, however, because hypermetabolism in dementia has been studied only to a limited extent [10, 59], with relatively little attention paid to subcortical metabolism [10]. In addition, differences in imaging protocols [60, 61], analytical pipelines [62, 63], and statistical thresholds [63, 64] further complicate such comparisons. To address this, we applied the same analysis to independent cohorts of patients with Alzheimer’s disease and frontotemporal dementia, as described in the Supplementary Materials. In neither cohort was hypermetabolism observed within the dentatorubrothalamic tract or mesencephalic tectum. These findings argue against the interpretation that the observed pattern reflects a normalization artefact and instead support the presence of absolute hypermetabolism.

Future research avenues should be twofold. On the one hand, evidence needs to be gathered to validate the hypothesized mechanism as the cause of the increased activity of the dentate nucleus, tectum mesencephali, and anterolateral thalamus. Particularly regarding the DRTT, research should focus on elucidating whether its role is compensatory or pathological. Additionally, quantification of increased metabolism in these regions in patients with DLB should be prioritized. This may help establish an objective early diagnostic biomarker for DLB, with potential value in distinguishing DLB from both healthy subjects and other challenging-to-diagnose causes of dementia. Specifically, future studies should perform formal sensitivity and specificity analyses of this marker to assess its utility as an adjunctive diagnostic tool. Given the broad clinical spectrum of DLB, future research should aim to delineate potential associations between specific clinical symptoms and distinct PET abnormalities.

## Conclusion

Glucose metabolism of the subcortical structures, as assessed by [^18^F]FDG PET brain imaging, sheds light on the potential role of connections between different subcortical nuclei in patients suffering from DLB. Particularly notable are the increased activities in the dentate nucleus and anterolateral thalamus, which can be explained by our hypothesis of involvement of the dentatorubrothalamic tract in DLB, and the mesencephalic tectum.

## Supplementary Information

Below is the link to the electronic supplementary material.Supplementary file1 (DOCX 423 KB)

## Data Availability

The datasets generated during and/or analysed during the current study are not available to ensure complete patient anonymity.
