# Supplementary material for: Assessing subcortical, brainstem and cerebellar metabolic patterns using [18F]FDG PET-CT imaging in dementia with Lewy bodies
Source: Eur J Nucl Med Mol Imaging. 2026 Jun 8;53(11):6167–83. doi: 10.1007/s00259-026-07925-z (PMC13526053; doi:10.1007/s00259-026-07925-z)
Supplement: Supplementary file 1 [file 259_2026_7925_MOESM1_ESM.docx]

### Alzheimer’s disease cohort

This group consists of patients with metabolic patterns and clinical characteristics of AD. Exclusion criteria were 1) patients younger than 18 years, 2) patients who refused to share anonymized retrospective data for research purposes, and 3) patients who showed metabolic changes suggestive of other neurodegenerative disorders (e.g., Lewy Body Dementia, Corticobasal Degeneration). Final diagnosis for all patients was based on a combination of clinical parameters, biomarkers in cerebrospinal fluid (when available, including tau, p-tau, and αβ amyloid), neuroimaging findings and disease progression over time in the years following the acquisition of the [18F]FDG-PET imaging used in this study. Clinical diagnosis was made by a board-certified neurologist or geriatrician based on the developed criteria for diagnosing Alzheimer’s dementia, as established by the National Institute on Aging and the Alzheimer’s Association (1).

This yielded a cohort of 23 patients with AD.

The imaging protocol, post-processing, SPM12 pipeline, control group, and statistical threshold (*P* < 0.01, FWE-corrected) were identical to those described in the main text for the DLB analysis.

### Frontotemporal dementia cohort

This group consists of patients with metabolic patterns and clinical characteristics of FTD. Exclusion criteria were 1) patients younger than 18 years, 2) patients who refused to share anonymized retrospective data for research purposes, and 3) patients who showed metabolic changes suggestive of other neurodegenerative disorders (e.g., Lewy Body Dementia, Corticobasal Degeneration). Final diagnosis for all patients was based on a combination of clinical parameters, biomarkers in cerebrospinal fluid (when available, including tau, p-tau, and αβ amyloid), neuroimaging findings and disease progression over time in the years following the acquisition of the [18F]FDG-PET imaging used in this study. Clinical diagnosis was made by a board-certified neurologist or geriatrician based on the developed criteria for diagnosing frontotemporal dementia, as established by the International Behavioural Variant FTD Criteria Consortium (1).

This resulted in 16 patients suffering from FTD.

The imaging protocol, post-processing, SPM12 pipeline, control group, and statistical threshold (*P* < 0.01, FWE-corrected) were identical to those described in the main text for the DLB analysis.

### Bibliography

1.	Loewenstein DSL, van Grinsven M, de Pont C, Dautzenberg PLJ, van Strien AM, Henssen D. Assessing the metabolism of the olfactory circuit by use of (18)F-FDG PET-CT imaging in patients suspected of suffering from Alzheimer's disease or frontotemporal dementia. Alzheimers Res Ther. 2024;16(1):241.
